# Supplementary material for: Moyamoya disease factor RNF213 is a giant E3 ligase with a dynein-like core and a distinct ubiquitin-transfer mechanism
Source: eLife. 2020 Jun 23;9:e56185. doi: 10.7554/eLife.56185 (PMC7311170; doi:10.7554/eLife.56185)
Supplement: Supplementary file 1. — Sequences were retrieved from NCBI non redundant protein database or from UniProt reference proteomes with the following accessions: Mus musculus (NCBI: ref|NP_001035094.2), Homo sapiens (Uniprot: sp|Q63HN8), Gallus gallus (NCBI: ref|XP_015151083.1), Xenopus laevis (Uniprot: tr|A0A1L8ETH7), Danio rerio (Uniprot: sp|A0A0R4IBK5); sequences were aligned with MAFFT version 7.427 (27), and visualized with Jalview26. Alpha helices (grey) and beta strands (black) are derived from the cryo-EM structure and shown on top. Residues with no structural data are indicated by a dashed line. At the bottom, positions with MMD mutations are marked by polygons, where green represents a low (<15), magenta an intermediate (<20) and red a high CADD score (>=20). Arrows indicate domain borders. Within the AAA+ domains, functional residues for nucleotide binding and hydrolysis are indicated by letters (A, Walker A; B, Walker B; S1, sensor I; S2, sensor II; RF, arginine finger). Regions IR3, IR5, and the E3-RING are enframed. [file elife-56185-supp1.pdf]

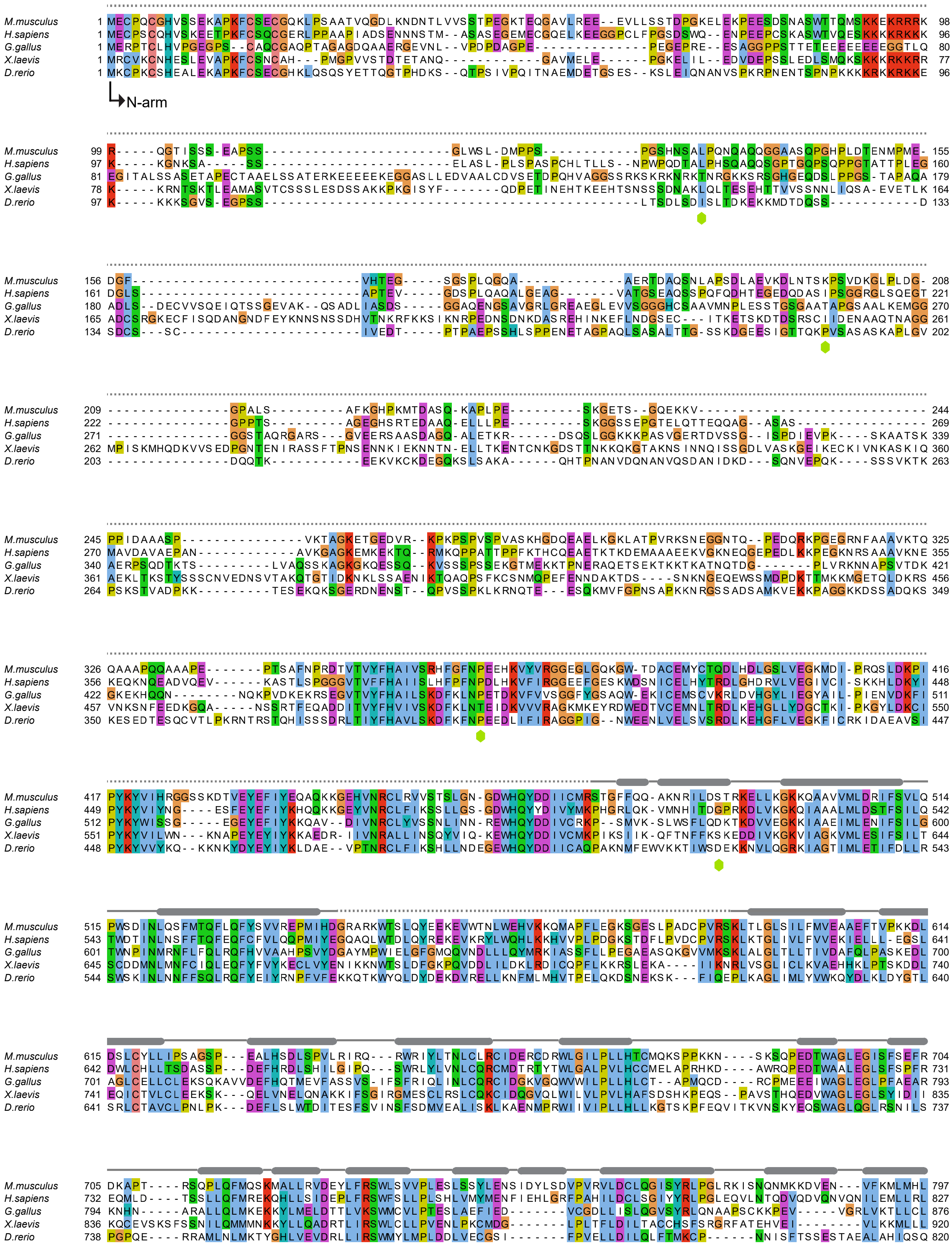

Extended Data 1- page 1

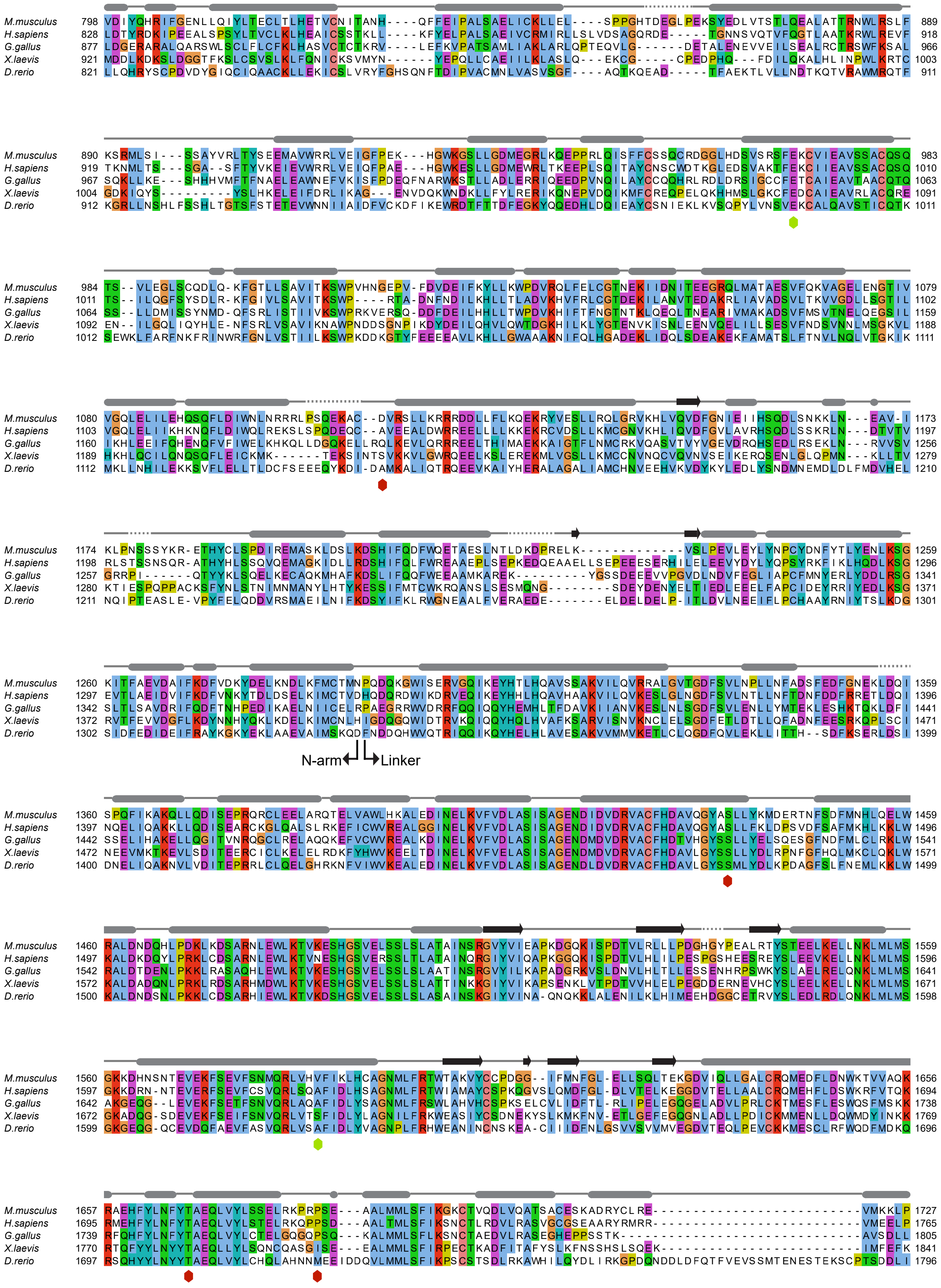

Extended Data 1- page 2

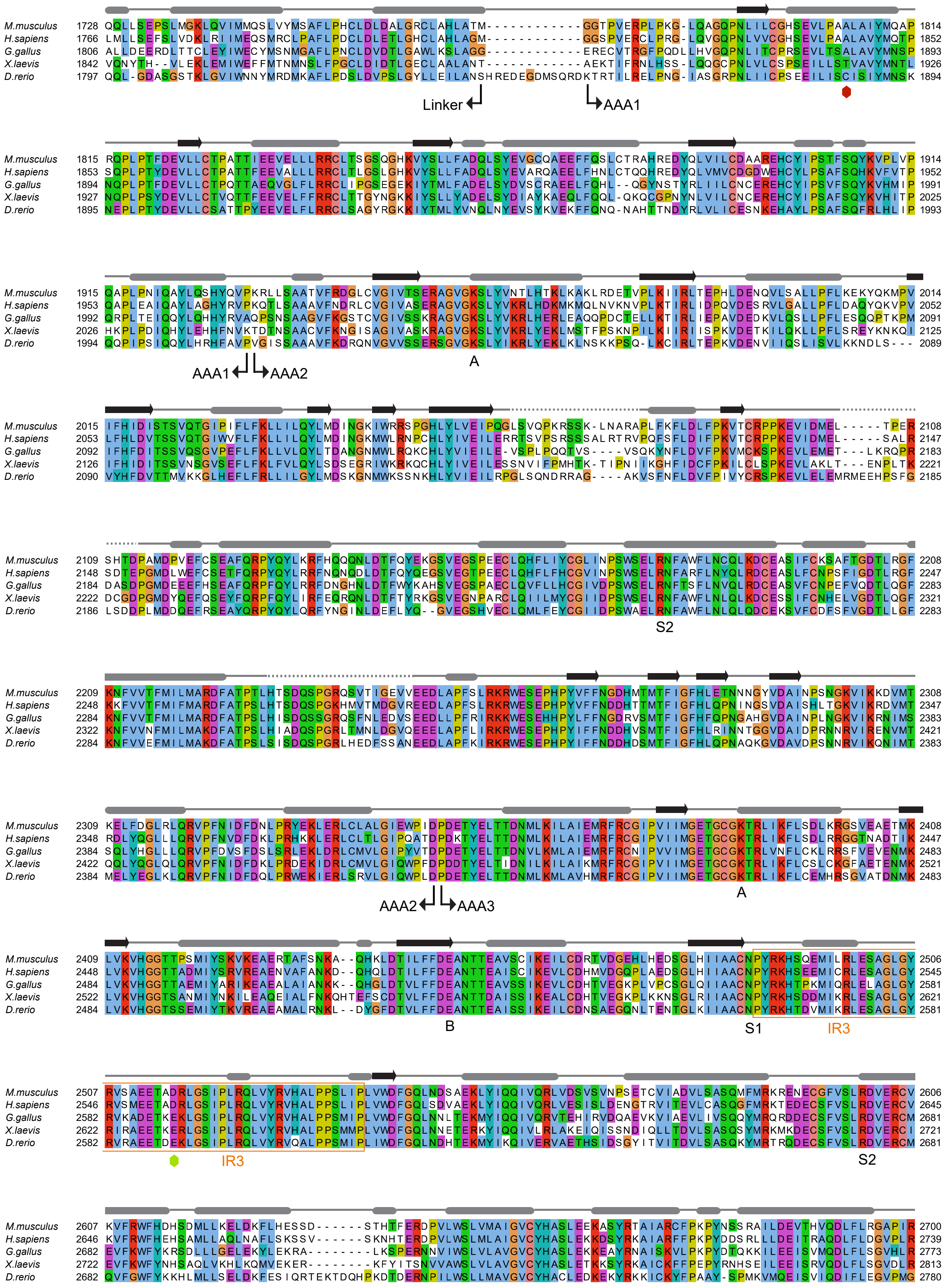

Extended Data 1- page 3

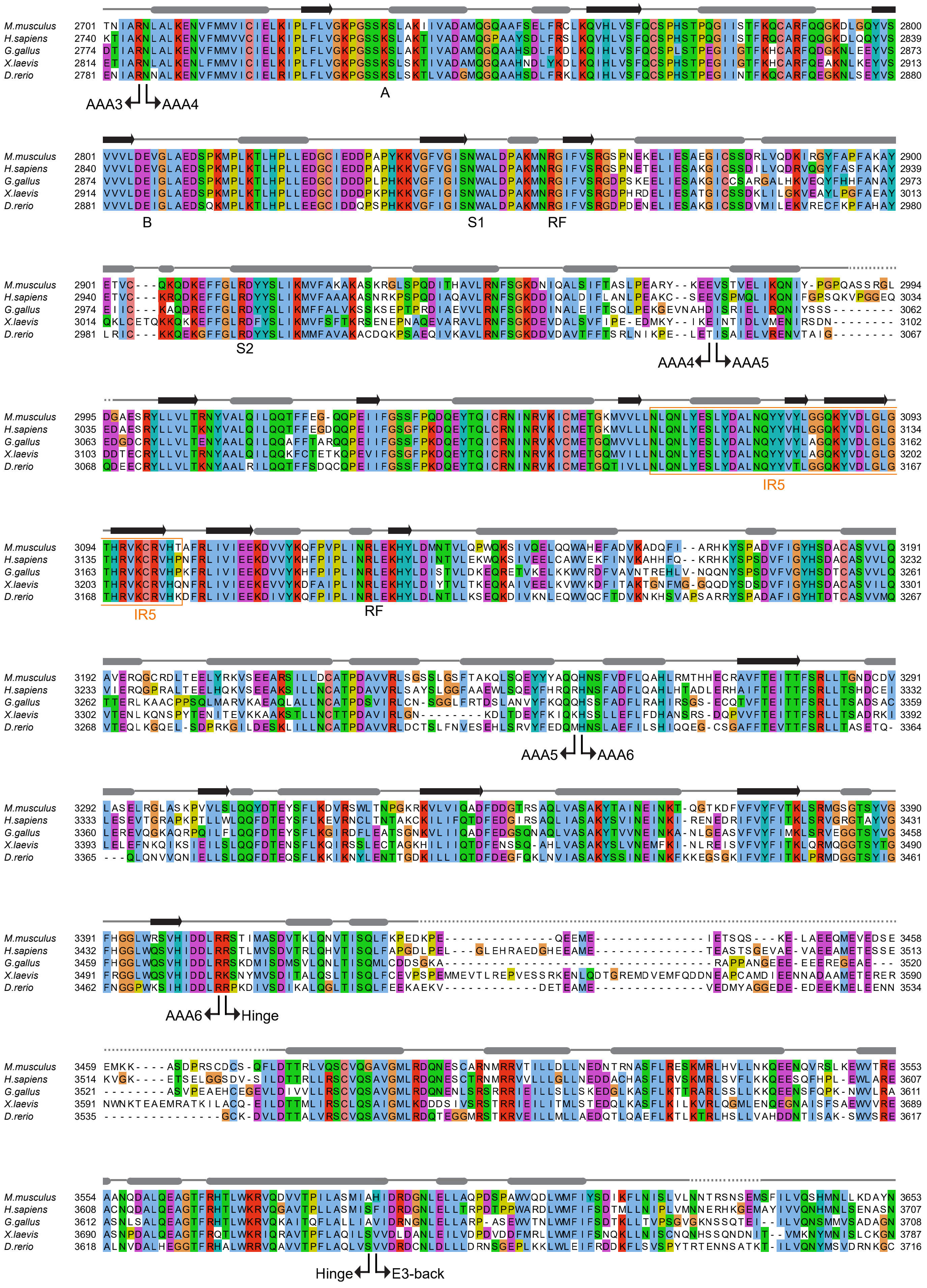

Extended Data 1- page 4

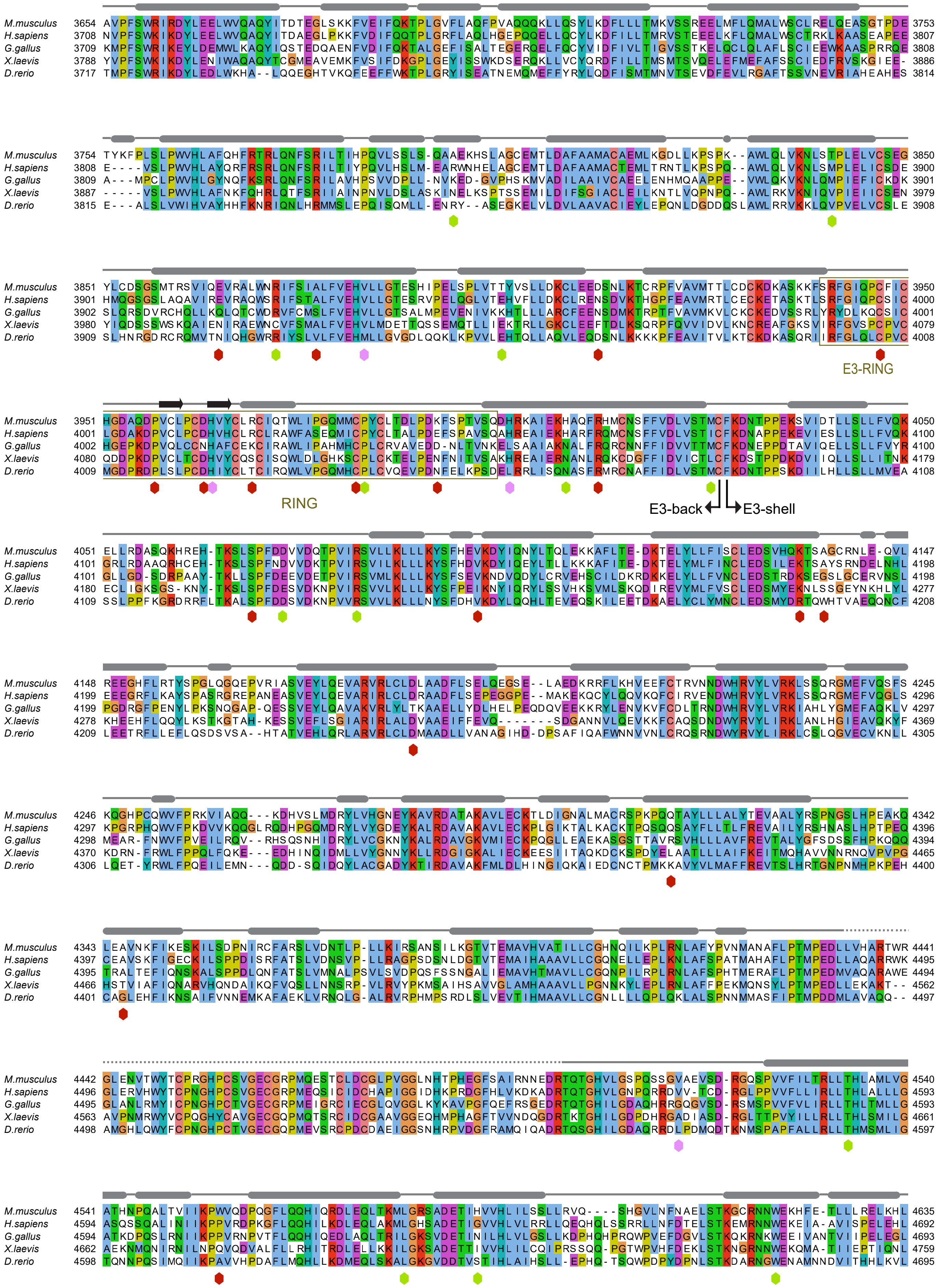

Extended Data 1 - page 5

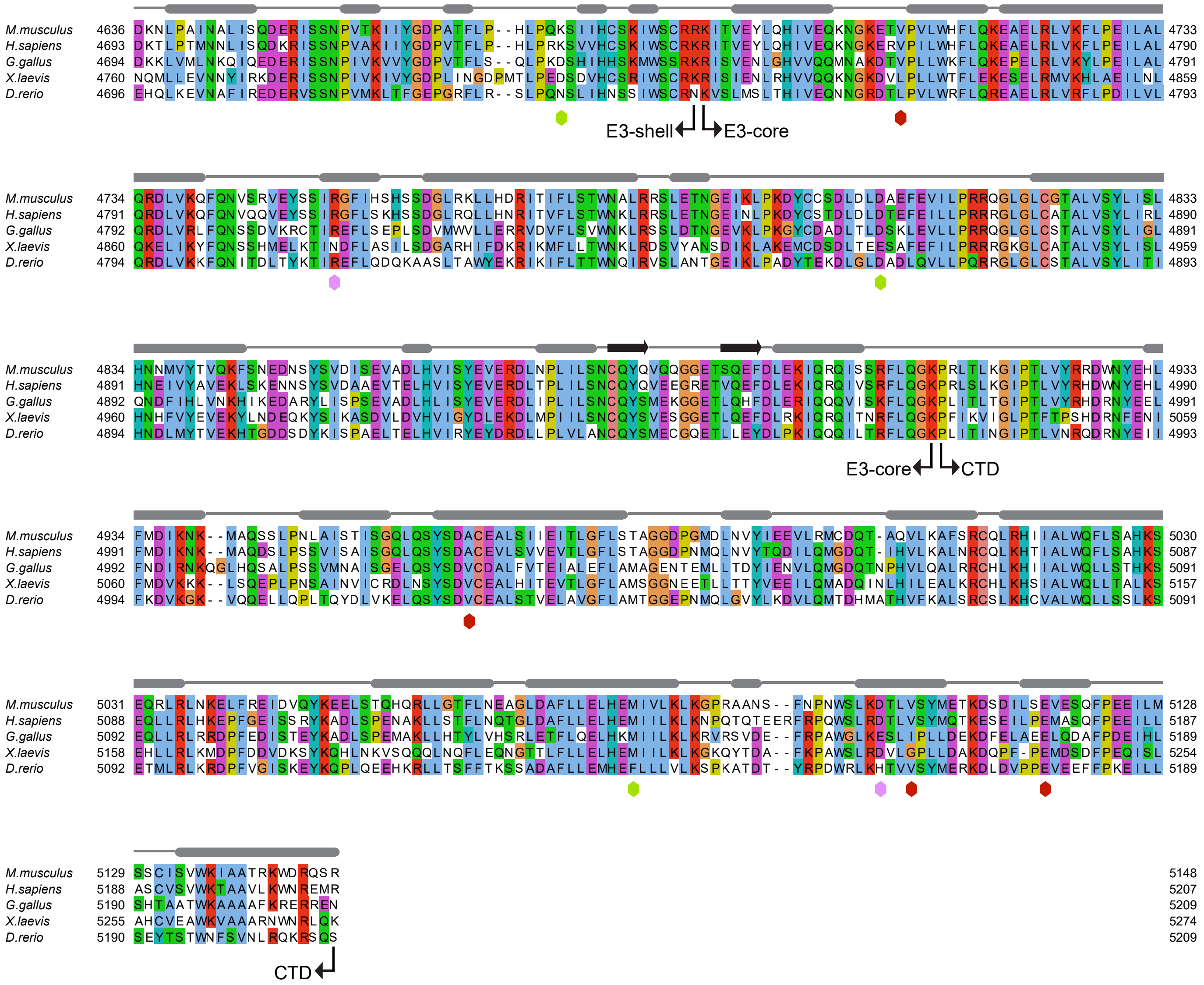

Multiple alignment of RNF213 orthologs. Sequences were retrieved from NCBI non redundant protein database or from UniProt reference proteomes with the following accessions: Mus musculus (NCBI: ref|NP_001035094.2), Homo sapiens (Uniprot: sp|Q63HN8), Gallus gallus (NCBI: ref|XP_015151083.1), Xenopus laevis (Uniprot: tr|A0A1L8ETH7), Danio rerio (Uniprot: sp|A0A0R4IBK5); sequences were aligned with MAFFT version 7.427 *(28)*, and visualized with Jalview *(27)*. Alpha helices (grey) and beta strands (black) are derived from the cryo-EM structure and shown on top. Residues with no structural data are indicated by a dashed line. At the bottom, positions with MMD mutations are marked by polygons, where green represents a low (<15), magenta an intermediate (< 20) and red a high CADD score (>= 20). Arrows indicate domain borders. Within the AAA+ domains, functional residues for nucleotide binding and hydrolysis are indicated by letters (A, Walker A; B, Walker B; S1, sensor I; S2, sensor II; RF, arginine finger). Regions IR3, IR5, and the E3-RING are enframed.
