# Supplementary material for: Moyamoya disease factor RNF213 is a giant E3 ligase with a dynein-like core and a distinct ubiquitin-transfer mechanism
Source: eLife. 2020 Jun 23;9:e56185. doi: 10.7554/eLife.56185 (PMC7311170; doi:10.7554/eLife.56185)
Supplement: Supplementary file 2. — For each variant, listed are the corresponding residue in mouse RNF213, whether the residue is conserved between mouse and human, the cDNA variants considered, and the CADD score for the least-scoring cDNA variant. The domain each residue belongs to is also indicated. CADD scores >= 20 are highlighted in red. Variant annotations refer to the RNF213 isoform 1 (RefSeq NM_001256071.3, human genome assembly GRCh38.p13). The lower panel shows the distribution per RNF213 module, highlighting the accumulation of MMD mutations in the composite E3 domain. [file elife-56185-supp2.docx]

| **Variant (Human)** | **Mouse residue** | **conserved** | **cDNA variants** | **CADD** | **domain** |
| --- | --- | --- | --- | --- | --- |
| **N-arm** |  | | | | |
| p.Cys118Arg | Trp114 |  | c.352T>C | 2 | N-hand |
| p.Leu133Met | Leu129 | yes | c.397C>A | 13 | N-hand |
| p.Ile209Asn | Lys197 |  | c.626T>A | 0 | N-hand |
| p.Pro395Leu | Pro364 | yes | c.1184C>T | 1 | N-hand |
| p.Gly517Arg | Ser489 |  | c.1549G>[A;C] | 1 | N-arm |
| p.Glu996Lys | Glu969 | yes | c.2986G>A | 10 | N-arm |
| p.Ala1135Val | Asp1112 |  | c.3404C>T | 22 | N-arm |
| **Linker** |  | | | | |
| p.Ser1474Phe | Ser1437 | yes | c.4421C>T | 23 | Linker |
| p.Ala1622Val | Val1586 |  | c.4865C>T | 2 | Linker |
| p.Thr1705Lys | Thr1667 | yes | c.5114C>A | 21 | Linker |
| p.Pro1721Leu | Pro1683 | yes | c.5162C>T | 24 | Linker |
| **AAA** |  | | | | |
| p.Ala1844Thr | Ala1806 | yes | c.5530G>A | 21 | AAA |
| p.Asp2554Glu | Asp2515 | yes | c.7662C>[A;G] | 3 | AAA |
| **E3** |  | | | | |
| p.Arg3846His | Ala3796 |  | c.11537G>A | 2 | E3-back |
| p.Met3891Val | Thr3841 |  | c.11671A>G | 2 | E3-back |
| p.Glu3915Gly | Glu3865 | yes | c.11744A>G | 23 | E3-back |
| p.Arg3922Gln | Arg3872 | yes | c.11765G>A | 10 | E3-back |
| p.Ala3927Thr | Ala3877 | yes | c.11779G>A | 26 | E3-back |
| p.Val3933Met | Val3883 | yes | c.11797G>A | 16 | E3-back |
| p.Glu3950Asp | Thr3900 |  | c.11850G>[C;T] | 9 | E3-back |
| p.Asn3962Asp | Asp3912 |  | c.11884A>G | 24 | E3-back |
| p.Cys3997Tyr | Cys3947 | yes | c.11990G>A | 21 | E3-RING |
| p.Pro4007Arg | Pro3957 | yes | c.12020C>G | 23 | E3-RING |
| p.Asp4013Asn | Asp3963 | yes | c.12037G>A | 24 | E3-RING |
| p.His4014Asn | His3964 | yes | c.12040C>A | 18 | E3-RING |
| p.Arg4019Cys | Arg3969 | yes | c.12055C>T | 26 | E3-RING |
| p.Cys4032Arg | Cys3982 | yes | c.12094T>C | 25 | E3-RING |
| p.Pro4033Leu | Pro3983 | yes | c.12098C>T | 0 | E3-RING |
| p.Glu4042Lys | Lys3992 |  | c.12124G>A | 22 | E3-RING |
| p.His4051Pro | His4001 | yes | c.12152A>C | 17 | E3-back |
| p.His4058Pro | His4008 | yes | c.12173A>C | 14 | E3-back |
| p.Arg4062Gln | Arg4012 | yes | c.12185G>A | 21 | E3-back |
| p.Ile4076Val | Met4026 |  | c.12226A>G | 10 | E3-back |
| p.Ser4118Phe | Ser4068 | yes | c.12353C>T | 21 | E3-shell |
| p.Asp4122Val | Asp4072 | yes | c.12365A>T | 11 | E3-shell |
| p.Arg4131Cys | Arg4081 | yes | c.12391C>T | 9 | E3-shell |
| p.Val4146Ala | Val4096 | yes | c.12437T>C | 24 | E3-shell |
| p.Lys4185Glu | Lys4135 | yes | c.12553A>G | 25 | E3-shell |
| p.Lys4185Thr | Lys4135 | yes | c.12554A>C | 25 | E3-shell |
| p.Ala4188Thr | Ala4138 | yes | c.12562G>A | 32 | E3-shell |
| p.Asp4237Glu | Asp4186 | yes | c.12711C>[A;G] | 27 | E3-shell |
| p.Gln4367Leu | Gln4313 | yes | c.13100A>T | 27 | E3-shell |
| p.Ala4399Thr | Ala4345 | yes | c.13195G>A | 25 | E3-shell |
| p.Val4567Met | Val4513 | yes | c.13699G>A | 15 | E3-shell |
| p.Thr4586Pro | Thr4533 | yes | c.13756A>C | 14 | E3-shell |
| p.Pro4608Ser | Trp4555 |  | c.13822C>T | 23 | E3-shell |
| p.Leu4631Val | Leu4578 | yes | c.13891C>G | 7 | E3-shell |
| p.Gly4640Ser | His4587 |  | c.13918G>A | 14 | E3-shell |
| p.Trp4677Leu | Trp4620 | yes | c.14030G>T | 8 | E3-shell |
| p.Lys4732Thr | Lys4675 | yes | c.14195A>C | 7 | E3-shell |
| p.Val4765Met | Val4708 | yes | c.14293G>A | 26 | E3-core |
| p.Arg4810Gly | Arg4753 | yes | c.14428A>G | 23 | E3-core |
| p.Arg4810Lys | Arg4753 | yes | c.14429G>A | 16 | E3-core |
| p.Asp4863Asn | Asp4806 | yes | c.14587G>A | 5 | E3-core |
| **CTD** |  | | | | |
| p.Ala5021Val | Ala4964 | yes | c.15062C>T | 23 | CTD |
| p.Met5136Ile | Met5079 | yes | c.15408G>[A;C;T] | 10 | CTD |
| p.Asp5160Glu | Asp5101 | yes | c.15480C>[A;G] | 18 | CTD |
| p.Val5163Ile | Val5104 | yes | c.15487G>A | 21 | CTD |
| p.Glu5176Gly | Glu5117 | yes | c.15527A>G | 21 | CTD |

| **start** | **end** | **name** | **mutation frequency** | **CADD >20** |
| --- | --- | --- | --- | --- |
| 1 | 1290 | N-arm | 3 in 1290 | 1 in 1290 |
| 1291 | 1774 | Linker | 4 in 484 | 3 in 484 |
| 1775 | 3405 | AAA | 2 in 1631 | 1 in 1631 |
| 3406 | 3588 | Hinge | 0 in 183 | 0 in 183 |
| **3589** | **4911** | **E3** | **41 in 1333** | **21 in 1333** |
| 3589 | 3939 | E3-back-a | 8 in 351 | 3 in 351 |
| 3940 | 3999 | E3-RING | 8 in 60 | 6 in 60 |
| 4000 | 4027 | E3-back-b | 4 in 28 | 1 in 28 |
| 4028 | 4688 | E3-shell | 17 in 661 | 9 in 661 |
| 4689 | 4911 | E3-core | 4 in 223 | 2 in 223 |
| 4912 | 5148 | CTD | 5 in 238 | 3 in 238 |
