# Supplementary material for: Moyamoya disease factor RNF213 is a giant E3 ligase with a dynein-like core and a distinct ubiquitin-transfer mechanism
Source: eLife. 2020 Jun 23;9:e56185. doi: 10.7554/eLife.56185 (PMC7311170; doi:10.7554/eLife.56185)
Supplement: Supplementary file 3. [file elife-56185-supp3.docx]

| Protein | wild type | | | | | R4753K | | | | |
| --- | --- | --- | --- | --- | --- | --- | --- | --- | --- | --- |
| EMDB accession number | EMD-10429 | | | | | EMD-10430 | | | | |
| PDB accession number | 6TAX | | | | | 6TAY | | | | |
| Imaging | | | | | | | | | | |
| Microscope | Titan Krios G3 | | | | | Titan Krios G3 | | | | |
| Detector | Gatan K2 | | | | | Gatan K2 | | | | |
| Acceleration Voltage (keV) | 300 | | | | | 300 | | | | |
| total exposure per movie (e/Å^2^) | 59.89 | | | | | 47.29 | | | | |
| number of frames per movie | 40 | | | | | 40 | | | | |
| pixel size (Å) | 1.04 | | | | | 1.04 | | | | |
| nominal magnification | 130kx | | | | | 130kx | | | | |
| defocus-range (µm) | –1.5 to –3.5 | | | | | –0.8 to –2.0 | | | | |
| collection software | SerialEM | | | | | SerialEM | | | | |
| micrographs collected | 4749 | | | | | 6561 | | | | |
| Reconstruction | | | | | | | | | | |
| refinement software | relion v3.0 | | | | | relion v3.0 | | | | |
| particles used in reconstruction | 374683 | | | | | 426312 | | | | |
| Box size | 352 | | | | | 352 | | | | |
| Symmetry imposed | C1 | | | | | C1 | | | | |
| Region | overall | AAA | E3 half1 | E3 half2 | N-arm | overall | AAA | E3 half1 | E3 half2 | N-arm |
| Accuracy of rotation (degrees) | 1.2 | 1.2 | 3.6 | 3.4 | 2.7 | 1.0 | 1.0 | 2.5 | 1.9 | 1.7 |
| Accuracy of translation (pixels) | 0.6 | 0.6 | 1.8 | 1.5 | 1.5 | 0.5 | 0.5 | 1.1 | 0.8 | 1.1 |
| Fourier completeness | 1.0 | 1.0 | 1.0 | 1.0 | 1.0 | 1.0 | 1.0 | 1.0 | 1.0 | 1.0 |
| Resolution at FSC=0.143 (Å) | 3.2 | 3.1 | 3.3 | 3.3 | 3.3 | 3.2 | 2.9 | 3.3 | 2.9 | 3.1 |
| Resolution at FSC=0.5 (Å) | 3.6 | 3.4 | 3.7 | 3.7 | 3.7 | 3.5 | 3.2 | 3.7 | 3.2 | 3.3 |
